# PI3K and ERK-Induced Rac1 Activation Mediates Hypoxia-Induced HIF-1α Expression in MCF-7 Breast Cancer Cells

**DOI:** 10.1371/journal.pone.0025213

**Published:** 2011-09-27

**Authors:** Jun Du, Rui Xu, Zhenzhen Hu, Yinhui Tian, Yichao Zhu, Luo Gu, Lei Zhou

**Affiliations:** 1 State Key Laboratory of Reproductive Medicine, Nanjing Medical University, Nanjing, China; 2 Department of Physiology, Nanjing Medical University, Nanjing, China; 3 Cancer Center, Nanjing Medical University, Nanjing, China; 4 Department of Cardiology, The First Affiliated Hospital of Nanjing Medical University, Nanjing, China; Florida International University, United States of America

## Abstract

**Background:**

Hypoxia-inducible factor 1 (HIF-1α) expression induced by hypoxia plays a critical role in promoting tumor angiogenesis and metastasis. However, the molecular mechanisms underlying the induction of HIF-1α in tumor cells remain unknown.

**Methodology/Principal Findings:**

In this study, we reported that hypoxia could induce HIF-1α and VEGF expression accompanied by Rac1 activation in MCF-7 breast cancer cells. Blockade of Rac1 activation with ectopic expression of an inactive mutant form of Rac1 (T17N) or Rac1 siRNA downregulated hypoxia-induced HIF-1α and VEGF expression. Furthermore, Hypoxia increased PI3K and ERK signaling activity. Both PI3K inhibitor LY294002 and ERK inhibitor U0126 suppressed hypoxia-induced Rac1 activation as well as HIF-1α expression. Moreover, hypoxia treatment resulted in a remarkable production of reactive oxygen species (ROS). N-acetyl-L-cysteine, a scavenger of ROS, inhibited hypoxia-induced ROS generation, PI3K, ERK and Rac1 activation as well as HIF-1α expression.

**Conclusions/Significance:**

Taken together, our study demonstrated that hypoxia-induced HIF-1α expression involves a cascade of signaling events including ROS generation, activation of PI3K and ERK signaling, and subsequent activation of Rac1.

## Introduction

Angiogenesis is a relatively early event in the progression of human cancer. Increased tumor angiogenesis correlates closely with tumor growth, hematogenous metastasis and poor cancer prognosis [1], [2]. It has been proposed that a wide variety of cytokines secreted by tumor cells such as vascular endothelial growth factor (VEGF) [3], basic fibroblast growth factor (bFGF), platelet-derived growth factor (PDGF) [4], erythropoietin [5], and insulin-like growth factor (IGF) [6] may stimulate angiogenesis.

Hypoxia, a characteristic feature of many human malignancies [7], [8], can stimulate the expression of numerous angiogenic factors by the induction of hypoxia-inducible factor-1 (HIF-1), which is a heterodimeric protein composed of HIF-1α and HIF-1β subunits. HIF-1β is known as the aryl hydrocarbon receptor nuclear translocater and is constitutively expressed in cells. HIF-1α expression is precisely regulated by cellular O_2_ concentration. Under normoxia, HIF-1α is found to bind to von Hippel-Lindau protein (pVHL), then ubiquitinated and degraded through the proteasomal pathway. However, hypoxia inhibits HIF-1α hydroxylation and allows its translocation to the nucleus, where it binds to HIF-1β to form an active complex and initiates the transcription of VEGF and other angiogenic factors [9], [10], [11]. Although the role of HIF-1α in angiogenesis has been reported, the upstream signaling events stimulating HIF-1α expression activated by hypoxia are still not well characterized.

Rac1 is one of the best-characterized member of small GTPases [12]. Intracellularly, the GTP-bound form of Rac1interacts with downstream effectors that control multiple cellular processes, including cytoskeleton organization [13], membrane trafficking [14], and gene transcription [15]. A growing body of evidence indicates that Rac1 can be activated by stress stimuli such as hypoxia [16]. The activation of Rac1 in hepatocellular carcinoma is correlated with VEGF expression and metastasis *in vivo* [17]. Impairment of ischemia-induced angiogenesis is also reported in Rac1 haploinsufficient mice [18]. Rac1 regulates the active NADPH oxidase complex assembly in most cells, and is recognized as a critical determinant of intracellular redox status [19]. Recent studies have shown that Rac1 is involved in the control of angiogenesis by inducing ROS production [20], [21]. In addition, the activation of phosphatidylinositol-3-kinase (PI3K) [22], [23] and extracellular signal-regulated kinase (ERK) [24], [25] has been identified as potent modulators of HIF-1α expression. ERK has also been identified as a potent modulator of Rac1 in different cancer cell types [26], [27]. In addition, a recent study showed that hypoxia-induced IL-18 increases Rac1 activity and HIF-1α expression in a PI3K/Akt-dependent manner [28]. Thus, it is worthwhile to explore whether PI3K and ERK signaling pathways are involved in hypoxia-induced Rac1 activation and HIF-1α induction in breast cancer cells. The results in the present study indicate that PI3K and ERK-mediated activation of Rac1 is involved in hypoxia-induced expression of HIF-1α, and hypoxia-induced ROS production initiates such signaling activation and HIF-1α expression.

## Results

### Hypoxia upregulates HIF-1α expression and activity *in vitro*

**Figure 1:**
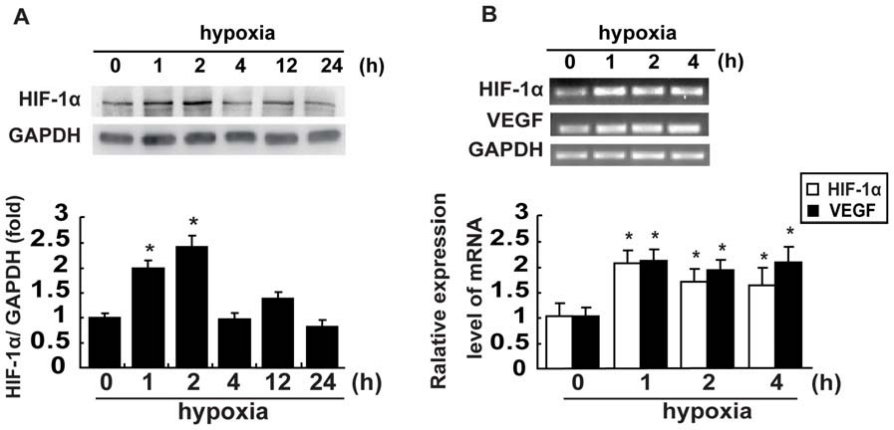
Hypoxia induces HIF-1α and VEGF expression in breast cancer cells. Confluent, serum-starved cells were incubated under normoxia or hypoxia for the indicated periods. (A) Total cellular proteins were extracted and analyzed for HIF-1α expression in MCF-7 cells by immunoblotting assays. A representative immunoblot of 5 independent experiments is shown here (upper panel). Protein densitometry was performed with the level of HIF-1α normalized against GADPH (lower panel). (B) Total cellular RNAs were extracted and analyzed for HIF-1α and VEGF mRNA expressions in MCF-7 cells as described under ‘Materials and Methods’. Data are presented as mean±SD of 5 independent determinations. *: *P*<0.05, referring to the difference between cells under hypoxia and those under normoxia.

It is widely accepted that HIF, and more specifically HIF-1α, is a central regulator of the cellular pathways under hypoxia [9]. To assess the effect of hypoxia on HIF-1α expression in breast cancer cells, MCF-7 cells were cultured under hypoxia for the indicated times. The results in Fig. 1A showed by immunoblotting assays that hypoxia induced an approximately 2-fold increase in HIF-1α levels in MCF-7 cells within 1 h. HIF-1α levels peaked at 2 h of hypoxia, and returned to the basal level at 4 h. We wished to further examine whether there were similar changes in HIF-1α expression at the transcriptional levels in MCF-7 cells. Our RT-PCR assays demonstrated that, indeed, the mRNA transcript levels of HIF-1α significantly increased in these cells in response to hypoxia, rapidly reaching the peak at 1 h and thereafter tapering off within 4 h (Fig. 1B). To determine whether this increase in HIF-1α expression was associated with an upregulation of HIF-1α transcriptional activity, we next analyzed the expression of the *VEGF* gene, a frequently used HIF-1α target gene [29]. We found that the VEGF mRNA transcript levels were significantly increased in MCF-7 cells under hypoxia (Fig. 1B).

### Hypoxia stimulates HIF-1α expression through activating Rac1

**Figure 2:**
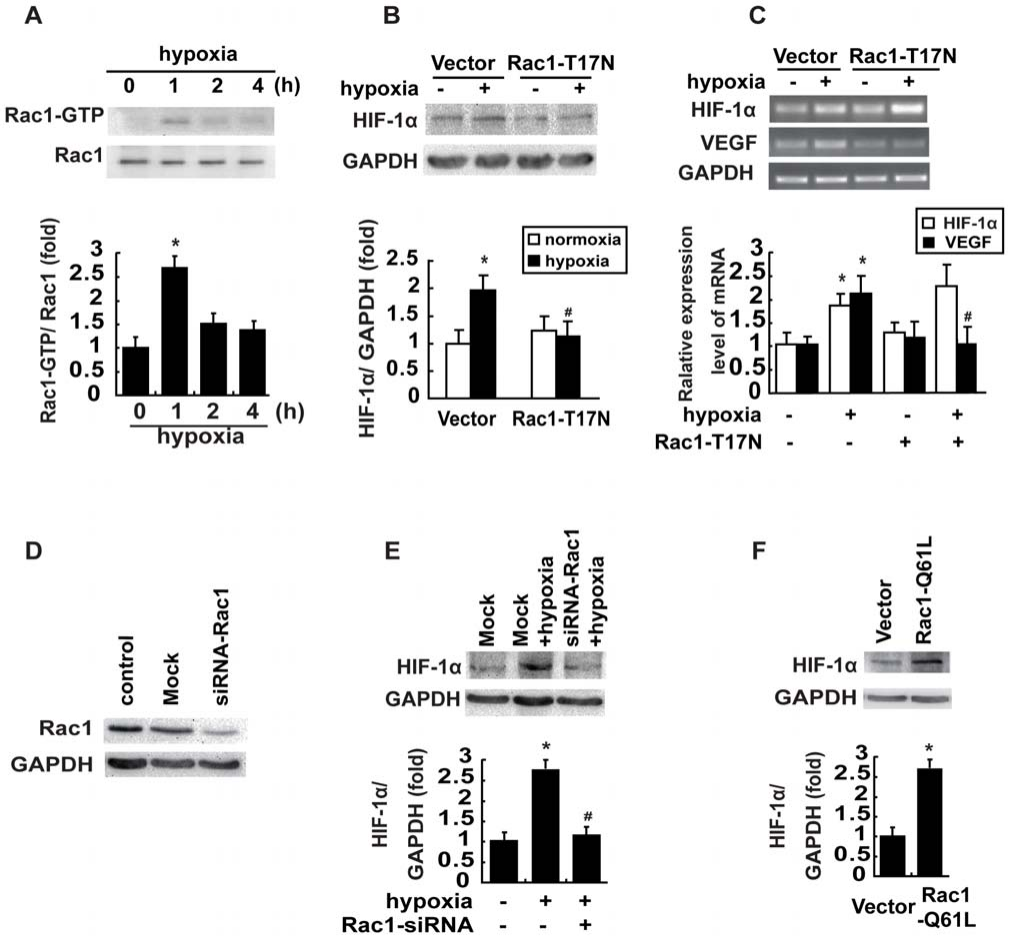
Rac1 activation is required for hypoxia-induced HIF-1α and VEGF expression. (A) Effect of hypoxia on Rac1 activation. Serum-starved MCF-7 cells were incubated under normoxia or hypoxia for the indicated periods. Cellular lysates were assayed for active Rac1 by pulldown assays as described in ‘Materials and methods’. Data are presented as mean ± SD of 5 independent determinations. (B&C) Overexpression of Rac1-T17N in cells inhibited hypoxia-mediated HIF-1α and VEGF expression. Cells transfected with empty vector or Rac1-T17N were grown under hypoxia for 2 h, HIF-1α protein (B), HIF-1α and VEGF mRNA expressions (C) were examined by immunoblotting or RT-PCR assays. (D) The effect of siRNA on the intracellular levels of Rac1. Total protein extracts from MCF-7 cells transfected with siRNA-Rac1 or scrambled siRNA (mock) were analyzed by Western blotting for Rac1. GAPDH was used a loading control. (E) Rac1 silencing inhibits HIF-1α expression. Cells transfected with empty vector or 60 nmol/L Rac1 siRNA were grown under hypoxia for 2 h, and HIF-1α protein were examined by immunoblotting assays. (F) Overexpression of Rac1-Q61L promoted HIF-1α expression. Cells were transfected with empty vector or Rac1-Q61L and HIF-1α expression was examined. *: *P*<0.05, referring to the difference between cells treated with and those without hypoxia. ^#^: *P*<0.05 (t-test), referring to the difference between the cells transfected with Rac1 siRNA or Rac1-T17N or Rac1-Q61L and the cells transfected with scrambled siRNA (mock) or empty vector.

Accumulating evidence has indicated that Rac1 can be activated by various stress stimuli such as hypoxia [16]. We wished to examine whether Rac1 influences HIF-1α expression in breast cancer cells under hypoxia. We first investigated whether hypoxia could regulate Rac1 expression and activation. Our pulldown assays revealed that hypoxia induced Rac1 activation with an early peak at 1 h, which then returned to the basal levels while the level of Rac1 in MCF-7 cells remained unmodified during 4 h of hypoxia (Fig. 2A). To determine whether hypoxia-induced HIF-1α expression is Rac1-dependent, we blocked Rac1 activity by transfecting these cells with Rac1 T17N, and examined HIF-1α expression during hypoxia. We found that, in cells transfected with the empty vector, HIF-1α expression was increased approximately 2 folds under hypoxia compared with their counterparts grown under normoxia. However, Rac1 T17N significantly attenuated hypoxia-induced increase in HIF-1α expression (Fig. 2B).

We further examined whether Rac1 regulated HIF-1α and VEGF at the transcriptional level. Our RT-PCR assays indicated that Rac1 T17N did not have any effect on the mRNA expression of HIF-1α, However, Rac1 T17N significantly attenuated hypoxia-induced increase in VEGF mRNA transcript levels (Fig. 2C). Additionally, we examined the effect of Rac1 on hypoxia-induced HIF-1α expressions by knocking down Rac1 expression with appropriate siRNAs in MCF-7 cells. We found that, compared with scrambled siRNA (mock), siRNA against Rac1 effectively reduced Rac1 expression in MCF-7 cells under normoxia (Fig. 2D). Under hypoxic conditions, siRNA against Rac1 noticeably attenuated hypoxia-induced increase in the protein levels of HIF-1α (Fig. 2E). We further examined whether Rac1 could activate HIF-1α. We transfected MCF-7 cells with either empty vectors or vectors encoding the active mutant of Rac1 Q61L. Our immunoblotting assays demonstrated that the active mutant of Rac1 Q61L markedly increased the protein levels of HIF-1α under normoxia (Fig. 2F). These results showed that, under hypoxia, Rac1 can regulate HIF-1α expression and in turn its transcriptional activities *in vitro*.

### Rac1 activation is required for hypoxia-stimulated nuclear translocation of HIF-1α in MCF-7 cells

**Figure 3:**
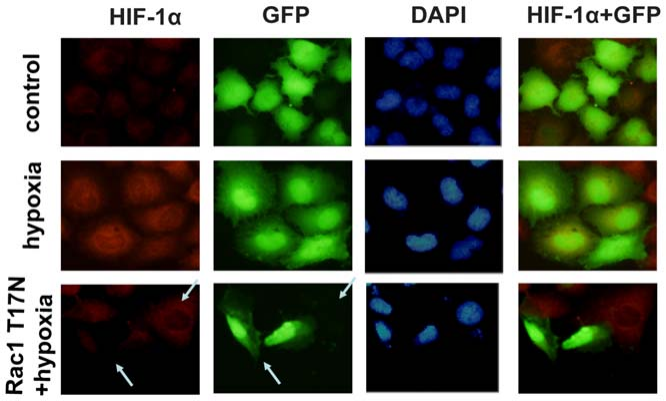
Rac1 activation is required for hypoxia-stimulated nuclear translocation of HIF-1α in MCF-7 cells. Cells transfected with GFP-Rac1-T17N or GFP (green) were grown under hypoxia for 2 h and examined HIF-1α expression (red) by immunofluorescence microscopy as described in ‘Materials and methods’. The cells were counter-stained with DAPI (blue). Images are representative of at least 3 independent determinations. The illustrations in the right side column show the merged images of HIF-1α and GFP fluorescence. Arrows in the left-bottom panel point to HIF-1α expression in cells transfected with or without GFP-Rac1-T17N. Cells transfected with GFP-Rac1-T17N showed weak HIF-1α (red) immunofluorescence staining compared with cells without transfection under hypoxia. Magnification, ×400.

To further determine whether hypoxia stimulates HIF-1α expression in a Rac1-dependent manner, we examined HIF-1α expression in MCF-7 cells under hypoxia by immunofluorescence microscopy. We found that HIF-1α showed weak expression and was localized in the cytoplasm of serum-starved MCF-7 cells under normoxia. At 2 h after exposure to hypoxia, HIF-1α abundance was obviously increased in both the cytoplasm and nucleus of these cells. However, Rac1 T17N noticeably attenuated the increase in HIF-1α expression and the subsequent nuclear translocation (Fig. 3).

### PI3K and ERK signaling mediate hypoxia-induced Rac1 activation and HIF-1α expression

**Figure 4:**
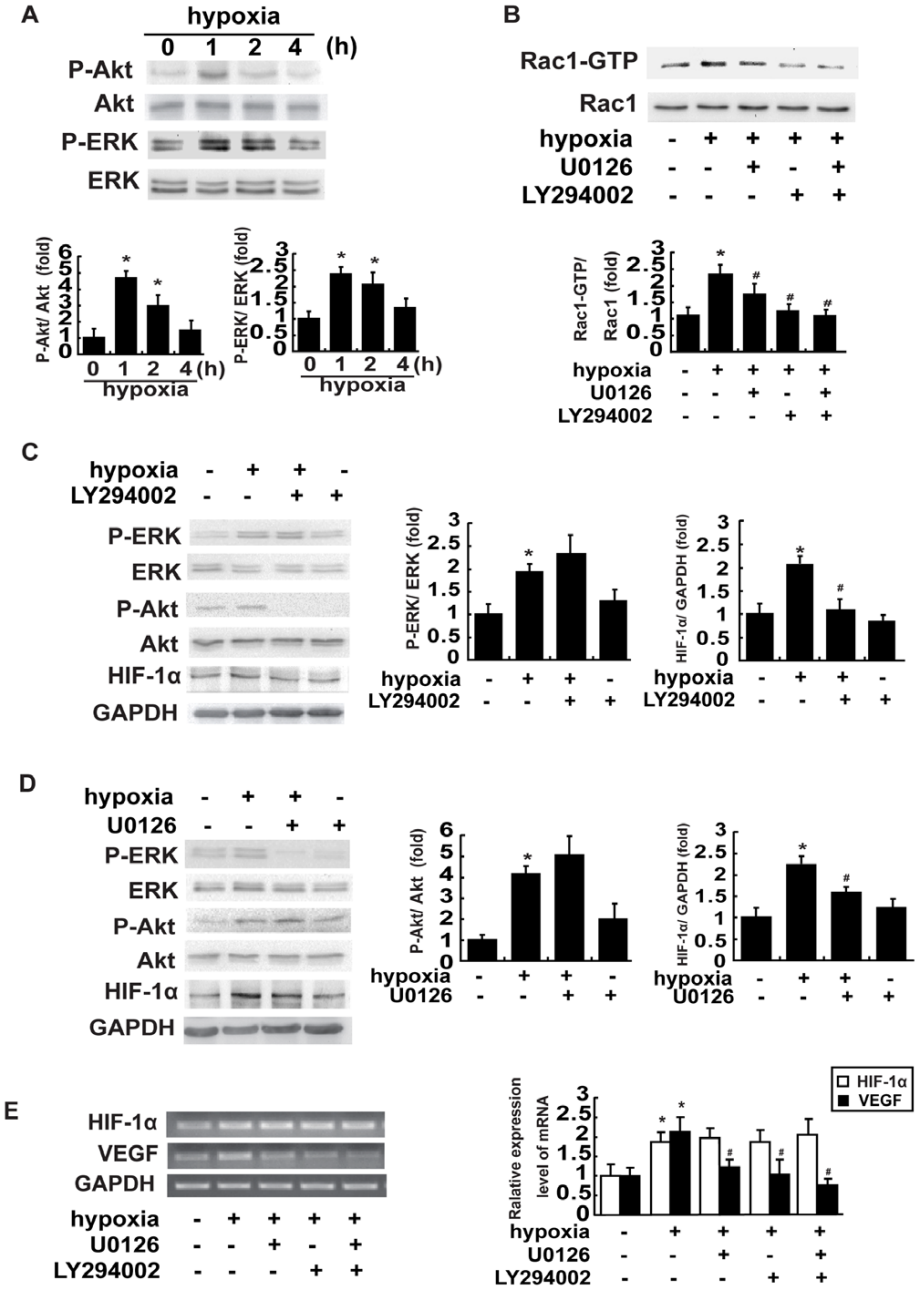
Hypoxia activates Rac1 via the PI3K and ERK pathway that is required for hypoxia-induced HIF-1α expression. (A) Effect of hypoxia on the activation of PI3K and ERK. Serum-starved MCF-7 cells were grown under normoxia or hypoxia for the indicated periods. Hypoxia-stimulated phosphorylation of Akt at Ser 473 and phosphorylation of ERK at Thr202/Tyr204 was determined as described in ‘Materials and methods’. (B) Hypoxia induced activation of Rac1 was dependent on PI3K and ERK. After treatment with 10 µM LY294002 or 10 µM U0126 for 1 h, the cells were incubated under hypoxia for 1 h and Rac1 activity was examined. (C) Hypoxia induced HIF-1α expression was dependent on PI3K. (D) Hypoxia induced HIF-1α expression was dependent on ERK. After treatment with 10 µM LY294002 (C) or 10 µM U0126 (D) for 1 h, MCF-7 cells were incubated under hypoxia for 1 h and were analyzed by PI3K, ERK or HIF-1α assays as described in ‘Materials and methods’. (E) Effects of LY294002 or U0126 on HIF-1α and VEGF mRNA expression in hypoxic cells. After pretreatment with 10 µM LY294002 or 10 µM U0126 for 1 h, the cells were incubated under hypoxia for 1 h and HIF-1α mRNA expressions was examined. Data are presented as mean ± SD of 5 independent determinations. *: *P*<0.05, referring to the difference between cells treated with and those without hypoxia. ^#^: *P*<0.05 (t-test), refers to the comparison of cells treated with hypoxia plus LY294002 or U0126 and the cells treated with hypoxia alone.

Previous report has shown that both PI3K and ERK can activate HIF-1α in laser-induced rat choroidal neovascularization [30]. It is possible that these proteins affect HIF-1α expression in hypoxic breast cancer cells. We first quantified the levels of activated PI3K and ERK in normoxic or hypoxic MCF-7 cells by immunoblotting analysis of phospho-Akt (Ser473), a well-accepted downstream target of PI3K, and phospho-ERK [31], [32]. We found that the levels of both phospho-Akt and phospho-ERK were significantly increased at 1 h after hypoxia, whereas the total protein amounts of Akt and ERK remained unaltered (Fig. 4A). To determine whether the PI3K and ERK signaling pathway is involved in hypoxia-induced Rac1 activation, we blocked PI3K activity by using LY294002 (PI3K inhibitor) or U0126 (MEK/ERK inhibitor) and examined Rac1 activity after hypoxia. The results showed that pretreatment with 10 µM LY294002 largely inhibited hypoxia-induced Rac1 activity (Fig. 4B). Rac1 activity was also partially inhibited by 10 µM U0126 (Fig. 4B). These results suggest that PI3K and ERK act as the upstream effector of Rac1 activation. We further investigated the effects of PI3K and ERK inhibitors on HIF-1α protein and mRNA expressions in MCF-7 cells. As expected, HIF-1α protein levels increased approximately 2-folds in hypoxic cells compared with those in normoxic cells. Pretreatment with 10 µM LY294002 or 10 µM U0126 largely abolished the stimulatory effect of hypoxia on HIF-1α expression. However, ERK phosphorylation was not inhibited by LY294002, and PI3K activity was not inhibited by U0126 (Fig. 4C and 4D). Furthermore, LY294002 or U0126 attenuated the level of activated VEGF mRNA transcripts, but did not change the activated HIF-1α mRNA transcription level under hypoxia (Fig. 4E), demonstrating that ERK and PI3K did not regulate HIF-1α expression during hypoxia at the transcriptional level.

### ROS generation is required for hypoxia-induced activation of PI3K, ERK, and Rac1 and HIF-1α expression

**Figure 5:**
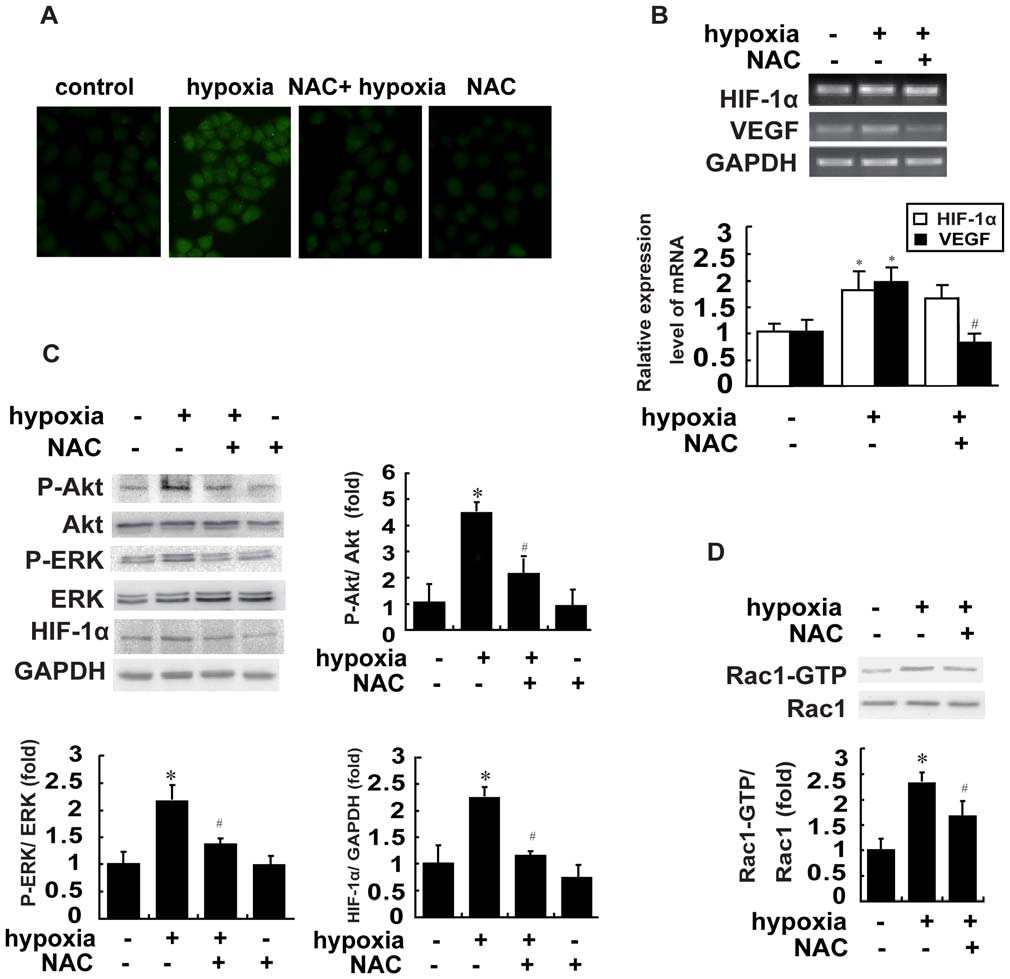
ROS generation is required for hypoxia-induced activation of PI3K, ERK and Rac1 and HIF-1α expression. (A) Representative micrographs of MCF-7 cells growing under normoxia or hypoxia without or with 2 mM NAC and stained with CM_2_-DCFHDA. (B) Effect of NAC on HIF-1α and VEGF mRNA expressions in hypoxic cells. After pretreatment with 2 mM NAC for 1 h, the cells were incubated under hypoxia for 1 h and HIF-1α and VEGF mRNA expressions was examined. (C&D) Effect of ROS inhibitor on hypoxia-stimulated PI3K, ERK and Rac1 activation and HIF-1α expression. After preincubation for 1 h with 2 mM NAC, cells were grown under hypoxia for 1 h and then cells were analyzed by Akt or ERK or Rac1 or HIF-1α assay as described in ‘Materials and methods’. Data are presented as mean ± SD of 5 independent determinations. *: *P*<0.05, referring to the difference between cells treated with and those without hypoxia. ^#^: *P*<0.05 (t-test), refers to the comparison of cells treated with hypoxia plus NAC and the cells treated with hypoxia alone.

**Figure 6:**
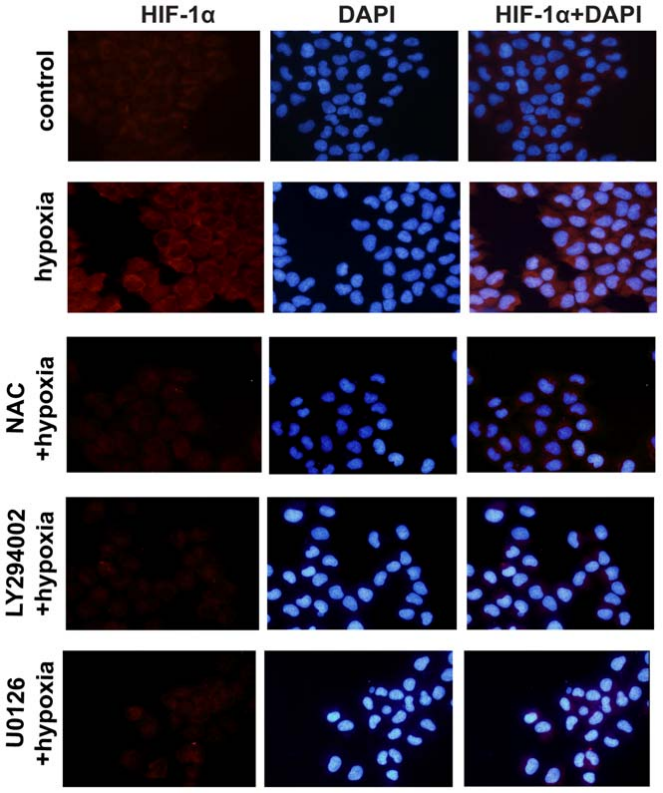
ROS inhibition reverses hypoxia-stimulated HIF-1α accumulation and nuclear translocation in MCF-7 cells. After pretreatment with 2 mM NAC, 10 µM LY294002 or 10 µM U0126 for 1 h, cells were grown under hypoxia for 2 h and stained for HIF-1α (red) as described in ‘Materials and methods’. Cells were counterstained with DAPI (blue). Images are representative of at least 3 independent determinations. The illustrations in the right side column show the merged images of HIF-1α and DAPI. Magnification, ×400.

It has been well documented that one of the main mediators of hypoxia in cells is ROS production [33], [34], and recent work has shown that ROS could regulate ovarian tumor angiogenesis and growth [35]. We examined the effect of hypoxia on ROS generation in cultured breast cancer cells by fluorescent staining with CM_2_-DCFHDA to measure endogenous H_2_O_2_ levels. The staining results revealed that H_2_O_2_ abundance was weak in serum-starved cultured cells, but increased dramatically under hypoxia. The role of ROS generation in hypoxia was further investigated by treatment of cells with N-acetyl-L-cysteine (NAC), a known scavenger of ROS. Two mM NAC prior to hypoxia diminished the production of H_2_O_2_ induced by hypoxia (Fig. 5A) with a corresponding reduction in hypoxia-induced PI3K, ERK and Rac1 activation. Furthermore, basal and hypoxia-stimulated HIF-1α expression was blocked markedly by pretreatment with NAC, while HIF-1α mRNA levels remained unchanged (Fig. 5B–D). To determine whether hypoxia stimulated ROS production was depended on NADPH oxidase activation, we treated cells with NADPH oxidase inhibitor diphenylene iodonium (DPI) before hypoxia. However, we found that the production of H_2_O_2_ by hypoxia was not altered in the presence of DPI (Figure S1). We also investigated whether ROS is involved in hypoxia-induced HIF-1α expression by immunofluorescence staining. As shown in Fig. 6, the inhibition of ROS by NAC before hypoxia prevented an increase in fluorescence intensity of HIF-1α protein in MCF-7 cells. Furthermore, we found that HIF-1α expression by hypoxia was inhibited by LY294002 or U0126. These results indicate that hypoxia can stimulate ROS generation in breast cancer cells, which acts as the upstream effector of PI3K and ERK in mediating Rac1 activation and HIF-1α expression in hypoxic MCF-7 cells.

## Discussion

HIF-1α is involved in tumor angiogenesis and metastasis by regulating genes involved in response to hypoxia. Clinical evidence showed that HIF-1α is associated with a worse prognosis in patients with invasive breast carcinoma [36]. In the present study, HIF-1α mRNA and protein expression was increased breast cancer cells undergoing hypoxia. The mRNA transcript levels of HIF-1α target gene *VEGF* were also upregulated by hypoxia. Therefore, hypoxia directly induces the expression and activation of HIF-1α in breast cancer cells, which is a critical step in tumor angiogenesis.

A primary observation in the present study is that hypoxia induced Rac1 activation in a time-dependent fashion in breast cancer cells. We further observed that hypoxia not only enhanced the protein level of HIF-1α in the cytoplasm and but also increased its redistribution from the cytoplasm to the nucleus. Rac1 is a known effector for pVHL suppression and linked to HIF-1α stabilization in hypoxia [37]. Rac1 activation promotes angiogenesis of various types of vascular endothelial cells [20], [38], and mediates hypoxia-stimulated breast cancer cell migration [39], [40]. A previous study has demonstrated that Rac1 activity corresponds with angiogenesis in hepatocellular carcinoma [17]. Similarly, our results showed that Rac1 Q61L increased HIF-1α protein expression in MCF-7 cells while preventing Rac1 activity by Rac1 T17N and siRNA Rac1 increased the HIF-1α protein and VEGF mRNA reduction under hypoxia, suggesting that hypoxia-induced Rac1 activity was responsible for the expression and activation of HIF-1α in breast cancer cells. Interestingly, we failed to observe any changes in HIF-1α mRNA transcript levels for Rac1 transfectants, indicating that Rac-induced HIF-1α expression in breast cancer cells is independent of mRNA regulation.

We next examined the potential activators for Rac1 in our system. PI3K and ERK are two major signaling pathways in regulating *in vivo* and *in vitro* angiogenesis [41], [42]. A previous study has demonstrated that PI3K and ERK signaling pathway induces the VEGF expression as well as promotes an increase in the development of choroidal neovascularization [30]. Shi YH. *et al.* also reported that bFGF induces HIF-1α activation and VEGF release in T47D breast cancer cell line involves PI3K/Akt and MEK1/ERK pathways [43]. Similar to these findings, our results revealed that hypoxia elevated not only the level of PI3K activity but also ERK phosphorylation in breast cancer cells. Furthermore, specific chemical inhibitors for these proteins cause the reduction of hypoxia-induced Rac1 activity and HIF-1α protein expressions. Therefore, it may be reasonable to speculate think that hypoxia-induced activation of Rac1 activation and enhanced HIF-1α protein stabilization is mediated through PI3K and ERK pathways. Although it is reported that ERK and PI3K could have crosstalk in some conditions [44], it is likely that hypoxia may act on two parallel and distinct signalling pathways to induce Rac1 activity, because ERK phosphorylation was not inhibited by PI3K inhibitor, and PI3K activity was not inhibited by ERK inhibitor either.

It remains unclear how PI3K causes the activation of Rac1 upon hypoxia. Akt is a crucial downstream effector of PI3K and is likely to be responsible for many biological consequences of PI3K activation. Previous studies demonstrated Akt could regulate the activation of GSK-3β, p70S6K1 and mTOR, and all of them were able to activate Rac1 [45], [46], [47], [48], suggesting that hypoxia stimulates Rac1 activation in breast cancer cells by activating PI3K/Akt signaling. It is not clear whether PI3K and ERK mediates HIF-1α protein expression through other ways in our system. Hypoxia has been known to promote ERK translocation to the nucleus, where ERK may exert part of its biological activity [49]. Previous report suggested that phosphorylation of p300 by the ERK increases HIF-1α transcriptional activity by increasing HIF-1/p300 complex formation [50]. However, our results showed that decreased activation of PI3K or ERK by inhibitor did not change the activated HIF-1α mRNA transcription level under hypoxia in MCF-7 human breast cancer cells. The role of other signaling molecules that function downstream of PI3K and ERK in controlling HIF-1α expression remains to be determined.

ROS plays a central role in the key intracellular signal transduction pathway for regulation of angiogenesis [51], [52]. An earlier report showed that thymosin beta-4-induced HIF-1α stabilization in human cervical tumor cells is ROS-dependent [53]. However, the mechanism whereby ROS influences HIF-1α expression has not been described. Some studies reported that overproduction of ROS induced HIF-1α accumulation via the PI3K/Akt-PKC-HDAC pathway [54]. In contrast, our results show that hypoxia increased the production of ROS in breast cancer cell line MCF-7. Furthermore, decreased activation of PI3K and Rac1 and reduced phosphorylation of ERK were observed after inhibition of ROS production, which correlated with ablation of HIF-1α expression, thus suggesting a dependency of ROS on the PI3K/Akt and ERK signaling pathways, as well as Rac1 activation and HIF-1α expression in MCF-7 cells.

It is noteworthy that Rac1 constitutes part of the structure of NADPH oxidase whereby it participates in the control of the intracellular ROS machinery [55]. However, in our study, decreased ROS production by NAC, a known scavenger of ROS, reduced Rac1 activation and HIF-1α expression. On the other hand, ROS production was not altered after abolition of NADPH oxidase activity by DPI (Figure S1). This result is consistent with findings showing that NAC inhibited Rac1 activity in response to LPS in macrophages [56]. Therefore, it may be reasonable that hypoxia-stimulated ROS generation in breast cancer cell line MCF-7 is independent of Rac1 activation.

ROS is supposed to act as an inhibitor of prolyl-4-hydroxylase (PHD), which mediates HIF-1α ubiquitination and degradation by hydroxylation of two specific proline residues of HIF-1α [57]. Therefore, it is not clear whether ROS induces HIF-1α expression through other ways in our system. Besides, how ROS induces PI3K or ERK activation in breast cancer cells in this study remains to be elucidated. Papaiahgari *et al.* suggested that epithelial growth factor receptor (EGFR) is the upstream regulator of both PI3K/Akt and MEK/ERK signaling pathways [58]. In accordance with previous studies, ROS notably induced EGFR activation in some cell types [58], [59], and we assume that ROS leading to EGFR activation possibly is related to PI3K and ERK activation during hypoxia. The mechanism whereby hypoxia regulates PI3K and ERK activation remains to be elucidated.

In summary, this study highlighted the role of Rac1 in the regulation of HIF-1α expression in hypoxic MCF-7 human breast cancer cells. PI3K and ERK-mediated activation of Rac1 is involved in hypoxia-induced production of HIF-1α. We have also found that hypoxia can stimulate ROS generation in breast cancer cells, which may serve as an important mediator for hypoxia to stimulate Rac1 activation through the activation of PI3K and ERK signaling pathway. The function and regulation of HIF-1α expression may be of major clinical importance and may provide new insights for the discovery of novel therapeutic targets.

## Materials and Methods

### Cells and plasmids

Human breast cancer cell line MCF-7 was obtained from the American Type Culture Collection (ATCC, Manassas, VA) and maintained at 37°C in high glucose Dulbecco's modified Eagle's medium (DMEM) (Gibco,Grand Island, NY) supplemented with 10% (v/v) fetal bovine serum (FBS) (Hyclone, Logan, UT), 100 units penicillin/ml, and 100 µg/ml streptomycin in a humidified atmosphere. For growth under hypoxia, the cells were incubated at 37°C in a modular chamber flushed with 1% O_2_, 5% CO_2_ and 94% N_2_. Cells were grown on coverslips for fluorescence staining and on plastic dishes for protein and mRNA extraction. Cells were made quiescent by serum starvation overnight followed by treatment. For drug studies, 10 µM LY294002 (Sigma, St. Louis, MO) or 10 µM U0126 (Alexis, Lausen, Switzerland) or 2 mM NAC (Sigma, St. Louis, MO) was added to the medium for 1 h before hypoxia.

pEGFP-C2 vector containing dominant negative Rac1-T17N insert or dominant positive Rac1-Q61L was kindly provided by Dr. Shoshana Ravid (The Hebrew University, Jerusalem, Israel). MCF-7 cells were transfected with either pEGFP-C2 or pEGFP-C2 expressing Rac1-T17N using Lipofectamine 2000 as instructed by the manufacturer (Invitrogen, Carlsbad, CA). The sequence of small interfering RNA (siRNA) for Rac1 was 5′-GUUCUUAAUUUGCUUUUCCTT-3′ and for the scrambled sequence 5′-GUACCGCACGUCAUUCGUAUC-3′ (GenePharma Co., Shanghai, China). Cells were grown until approximately 60% confluent and then transfected with Rac1 siRNA or scrambled siRNA using Lipofectamine 2000 as instructed by the manufacturer.

### Western blotting and immunofluorescence studies

Cellular lysates and immunoblotting were performed as previously depicted [60]. The following antibodies were used: rabbit anti-Akt antibody, rabbit anti-phospho-Akt (Ser473) antibody, rabbit anti-HIF-1α antibody (Cell Signaling Technology, Beverly, MA), rabbit anti-ERK antibody and anti-phospho-ERK (Thr202/Tyr204) antibody (Santa Cruz Biotechnology, Santa Cruz, CA), and mouse anti-GAPDH antibody (Chemicon, Temecula, CA). Digital images of the immunoblotts were obtained with a Chemidoc XRS and analyzed using the image analysis program Quantity One (Bio-Rad, Hercules, CA). For immunofluorescence studies, cells were fixed in 3.7% paraformaldehyde in phosphate buffered saline (PBS) for 30 min, permeabilized in 0.1% Triton X-100 and blocked in PBS containing 1% bovine serum albumin (BSA) for 1 h at room temperature. The cells were incubated with rabbit anti-HIF-1α antibody (Novus, Littleton, Colorado) for 2 h followed by incubation with rhodamine conjugated anti-rabbit antibody for 1 h at room temperature within a moist chamber. Following wash with PBS, the cover slips were mounted on glass slides with DAPI Fluoromount G (Southern Biotech, Birmingham, AL). Images were collected using an Olympus BX51 microscope coupled with an Olympus DP70 digital camera.

### Reverse transcription polymerase chain reaction (RT-PCR)

Total RNAs were isolated with the TRIzol reagent according to the manufacturer's protocol (Invitrogen, Carlsbad, CA). Then, cDNA was synthesized using the SuperScript First Strand Synthesis System (Invitrogen) and amplified by polymerase chain reaction (PCR) using the following primers: GAPDH: 5′-TGAACGGGAAGCTCACTGG-3′ (sense) and 5′-TCCACCACCCTGTTGCTGT A-3′ (antisense); HIF-1α: 5′-ACAAGTCACCACAGGACA-3′ (sense) and 5′-AGGGAGAAAATCAAGTCG-3′ (antisense); VEGF: 5′-CGGGAACCAGAT CTCTCACC-3′ (sense) and 5′-AAAATGGCGAATCCAATTCC-3′ (antisense). The PCR for GAPDH was performed in 26 cycles at 95°C for 30 s, 58°C for 30 s, and 72°C for 30 s, for HIF-1α in 28 cycles at 95°C for 30 s, 46.6°C for 30 s, and 72°C for 40 s, and for VEGF in 28 cycles at 95°C for 30 s, 56°C for 30 s, and 72°C for 30 s. The PCR products were resolved by electrophoresis on 1% agarose. Images of electrophoresis were taken using the ChemiDOC XRS Imaging system (Bio-Rad, Hercules, CA).

### Pulldown assays

Rac1 activity was measured as previously depicted [61]. In brief, equal volumes of total cellular protein were incubated with GST-RBD (a gift from James E Casanova, University of Virginia, VA) beads captured on MagneGST Glutathione Particles (Promega, Madison, WI) for 1 h at 4°C. The particles were then washed three times with washing buffer containing 4.2 mM Na_2_HPO_4_, 2 mM KH_2_PO_4_, 280 mM NaCl, and 10 mM KCl (pH 7.2), resuspended in 2×SDS sample buffer and subjected to immunoblotting analysis by using a mouse anti-Rac1 antibody (Upstate Biotechnology, Lake Placid, NY).

### Intracellular ROS staining

For intracellular ROS staining, 1×10^5^ MCF-7 cells were seeded on a coverslip placed in a 6-well plate and incubated overnight. After cells were treated with appropriate inhibitors and stimuli as detailed elsewhere in the text, they were stained with 5 µM 2′,7′-dichlorofluorescein diacetate (CM_2_-DCFHDA) (Invitrogen) for 15 min at 37°C, washed with PBS three times, and fixed with 4% formaldehyde. After wash with PBS, the cover slips were mounted on glass slides with Fluoromount (Sigma). Images were collected using an Olympus BX51 microscope coupled with an Olympus DP70 digital camera.

### Statistical analysis

Statistical analysis was carried out using the SPSS software. Student's *t* test was used to analyze the differences between two groups. When comparisons between multiple groups were carried out, one-way ANOVA followed by SNK tests were employed. Statistical significance was considered at *P*<0.05.

## Supporting Information

Figure S1 **Effect of DPI on hypoxia-stimulated ROS production.** After pretreatment with 10 µM DPI for 1 h, cells were grown under hypoxia for 1 h and stained with CM_2_-DCFHDA. Images are representative of at least 3 independent determinations. Magnification, ×400.
(TIF)
