# Supplementary figures and images for: PI3K and ERK-Induced Rac1 Activation Mediates Hypoxia-Induced HIF-1α Expression in MCF-7 Breast Cancer Cells
Source: PLoS One. 2011 Sep 27;6(9):e25213. doi: 10.1371/journal.pone.0025213 (PMC3181265; doi:10.1371/journal.pone.0025213)

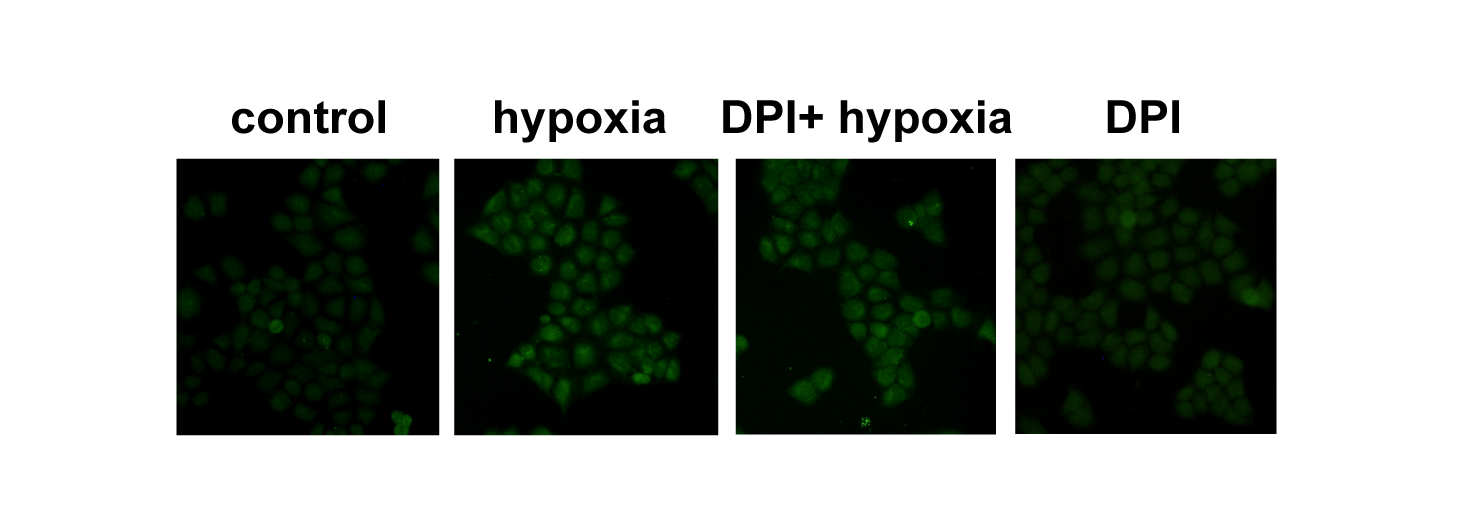

Supplement: Figure S1 — Effect of DPI on hypoxia-stimulated ROS production. After pretreatment with 10 µM DPI for 1 h, cells were grown under hypoxia for 1 h and stained with CM2-DCFHDA. Images are representative of at least 3 independent determinations. Magnification, ×400. (TIF) [file pone.0025213.s001.tif]
